# Supplementary material for: Clinically translatable mitochondrial gene therapy in muscle using tandem mtZFN architecture
Source: EMBO Mol Med. 2025 Apr 9;17(6):1222–37. doi: 10.1038/s44321-025-00231-5 (PMC12163086; doi:10.1038/s44321-025-00231-5)
Supplement: Supplementary file 1 — Appendix [file 44321_2025_231_MOESM1_ESM.pdf]

Appendix

Clinically translatable mitochondrial gene therapy in muscle using tandem mtZFN architecture

Pavel A Nash *et al.*

Table of contents

Appendix Figures

Appendix Figure S1. Assessment of mtZFN-AAV genome fidelity – page 2

Appendix Figure S2. Assessment of off-target effects of tandem mtZFN within murine heart nuclear DNA following delivery by AAV9.45 in vivo – page 3

Appendix Figure S3. Western blot analysis of intramuscular injection delivery to quadriceps of mice – page 4

Appendix Figure S4. Primary Northern blot data and quantifications for main text figure – page 5

Appendix Figure S5. Inflammatory and innate immune responses to muscle gene therapy upon local administration – page 6

Appendix Figure S6. InDel frequencies at nDNA off-target regions in muscle following AAVMYO treatment – page 7

Appendix Figure S7. Comparison of AAV dosages in murine mtZFN experiments with human ZFN clinical trials – page 8

Appendix supplementary data:

Alignment of MTM25(+)-T2A-WTM1(-) nucleotide sequence with MTM25-T2A-WTM1 after re-coding of WTM1. N.B. WTM1 FLAG tag replaced with HA during re-coding – page 9

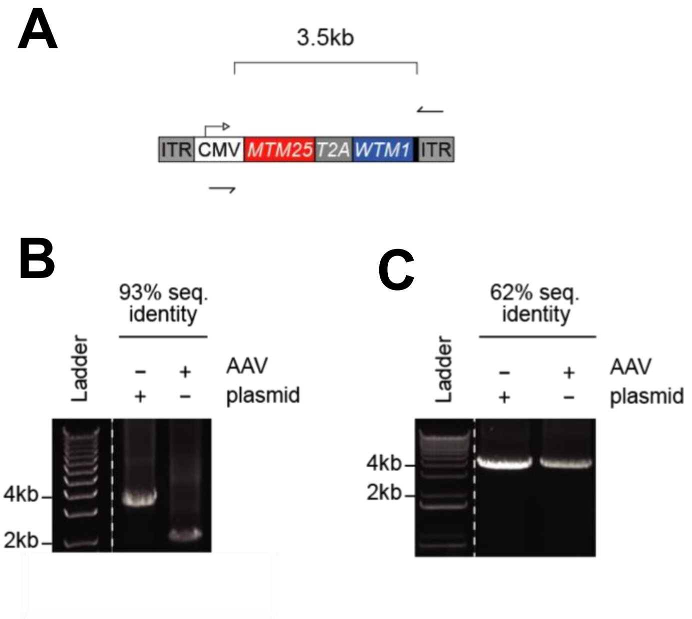

Appendix Figure S1. Assessment of mtZFN-AAV genome fidelity

(**A**) Schematic of tandem mtZFN transgene in both plasmid and resulting AAV genome. CMV F and BGH R primer binding sites are indicated by black arrows. (**B**) PCR analysis of the MTM25-T2A-WTM1 transgene, with 93% sequence identity between the MTM25 and WTM parts, from template plasmid and viral DNA following encapsidation into AAV9.45 using CMV F and BGH R primers. (**C**) PCR analysis of the recoded MTM25-T2A-WTM1 transgene, with 62% sequence identity between the MTM25 and WTM parts, from template plasmid and viral DNA following encapsidation into AAV 9.45 using CMV F and BGH R primers.

This analysis reveals large deletions of the MTM25-T2A-WTM1 transgene following viral genome replication and encapsidation (A, B), that are ameliorated by extensive recoding of the WTM1 portion of the transgene, eliminating a substantial proportion of DNA sequence homology between MTM25 and WTM1 and reducing the length of any given tract of homologous DNA sequence from > 200 nt to no more than 14 nt (C).

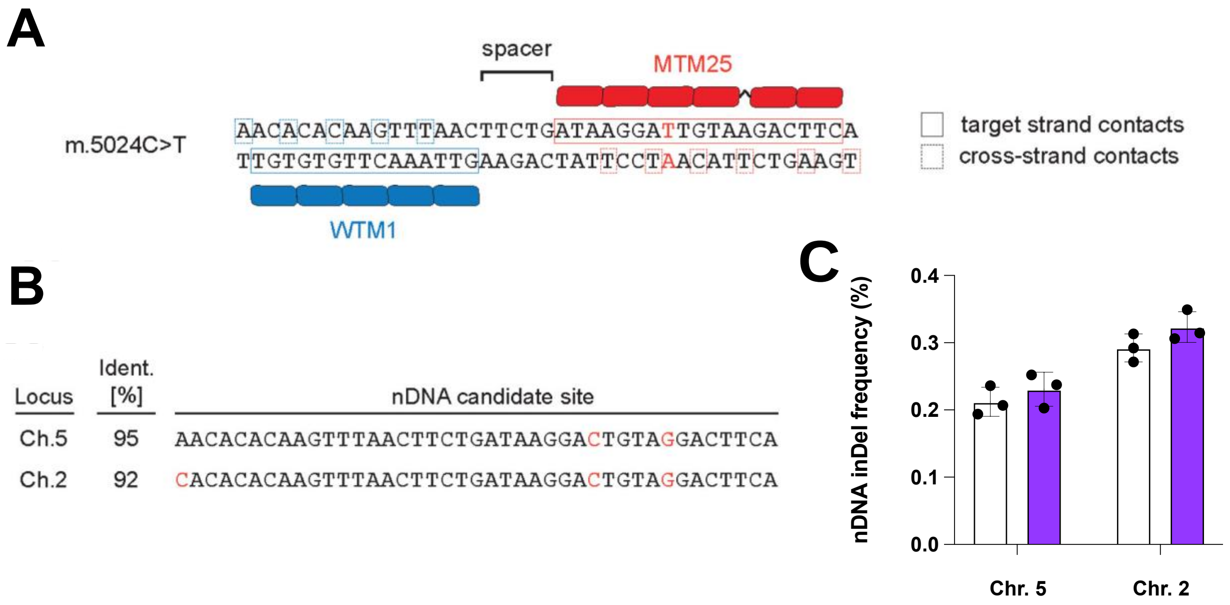

Appendix Figure S2. Assessment of off-target effects of tandem mtZFN within murine heart nuclear DNA following delivery by AAV9.45 *in vivo*

(**A**) Schematic of mtZFN binding at the m.5024C>T mtDNA target site, indicating target strand (solid line box) and cross-strand contacted bases (dashed line box) in mtDNA homologous loci in nDNA. (**B**) Locus, degree of homology (Ident.), sequence and InDel frequency at two nDNA regions of very high sequence homology with the mtZFN target site in mtDNA. InDel frequency within a 100 nt range of the target site was assessed, as described previously by Gammage et al. (**C**) Total DNA from hearts of three animals injected with 5E11 vg/mouse MTM25-T2A-WTM1-AAV9.45 (shown in purple) and three control animals (shown in white) were amplified by PCR and subjected to ultra-deep sequencing (>100,000 read depth). Black dots indicate individual animals. Error bars indicate S.E.M. Measure of center is the mean.

No differences in InDel frequency were detected at either nDNA site in controls or AAV-treated animals that demonstrate large shifts in heteroplasmy (Fig. 3B, main text).

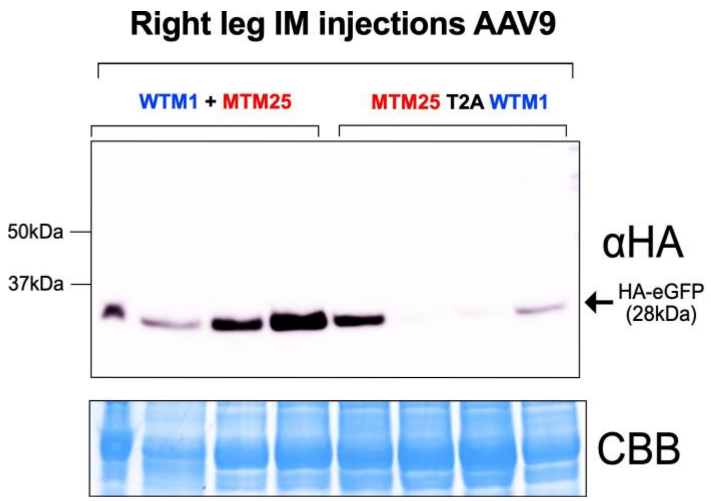

Appendix Figure S3. Western blot analysis of intramuscular injection delivery to quadriceps of mice

The HA tag on the eGFP was detected by immunoblotting against HA on the right legs of mice injected locally.

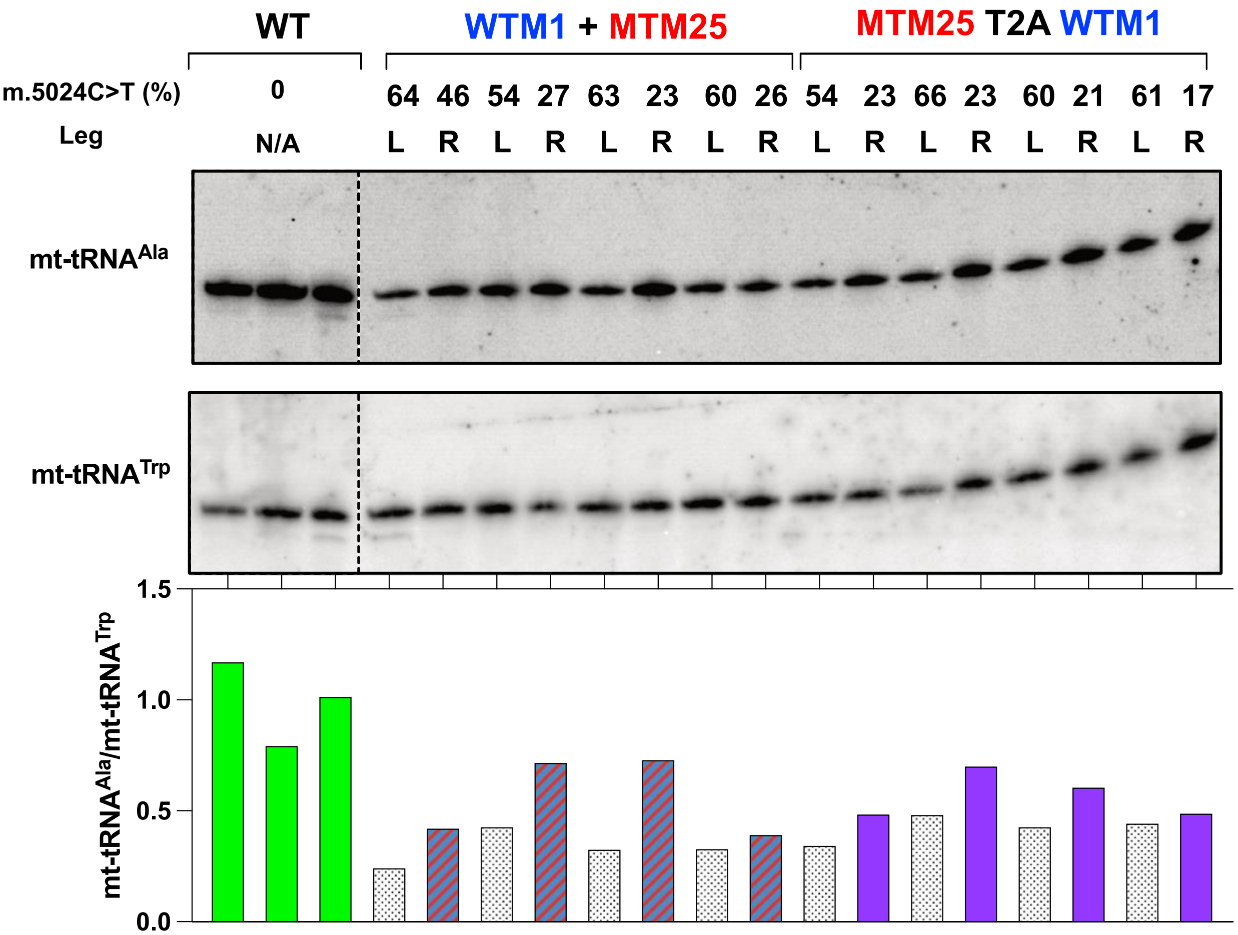

Appendix Figure S4. Primary Northern blot data and quantifications for main text figure

Northern blot of left and right legs from intramuscular experiments compared to wild type C57BL/6J skeletal muscle. Heteroplasmy values of both left (L) and right (R) legs are indicated. Mt-tRNATrp was used as control for mt-tRNA abundance with ratios digitally quantified and displayed for each sample. Dotted white indicates left vehicle treated legs, green indicates wild type, blue and red indicates separate administration and purple indicates tandem.

A

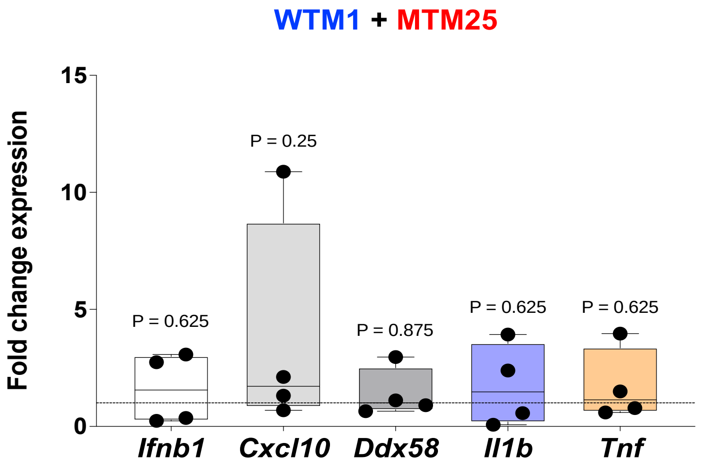

B

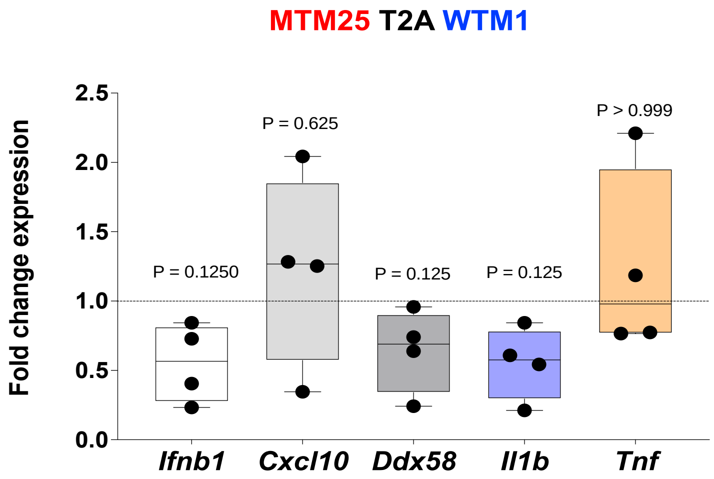

Appendix Figure S5. Inflammatory and innate immune responses to muscle gene therapy upon local administration

RT-qPCR measurements of transcripts pertaining to innate immunity and inflammation 65 days post-rAAV-administration for both separate (A) and tandem administration (B). Four mice were used for each condition. Samples for both separate and tandem administration were performed in technical quadruplicates and ratios calculated between right versus left leg. Boxplots show Interquartile range as the upper and lower bound of the box, whiskers show minimum and maximum measurements. Statistics were calculated using Wilcoxon signed rank test.

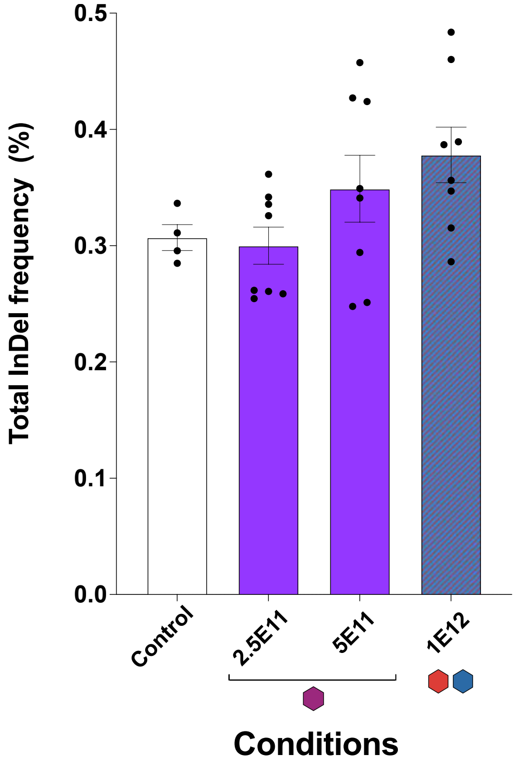

Appendix Figure S6. InDel frequencies at nDNA off-target regions in muscle following AAVMYO treatment

Summary data showing the percentage of NGS reads containing insertions or deletions at two regions in nDNA (chromosomes 2 and 5) in skeletal muscle samples with near perfect homology to the mitochondrial target window of the mtZFNs. No significant differences in InDel detection were observed when performing one-way ANOVA and Dunnett's comparison to the control samples. Each condition had 8 mice with 4 mice used as control.

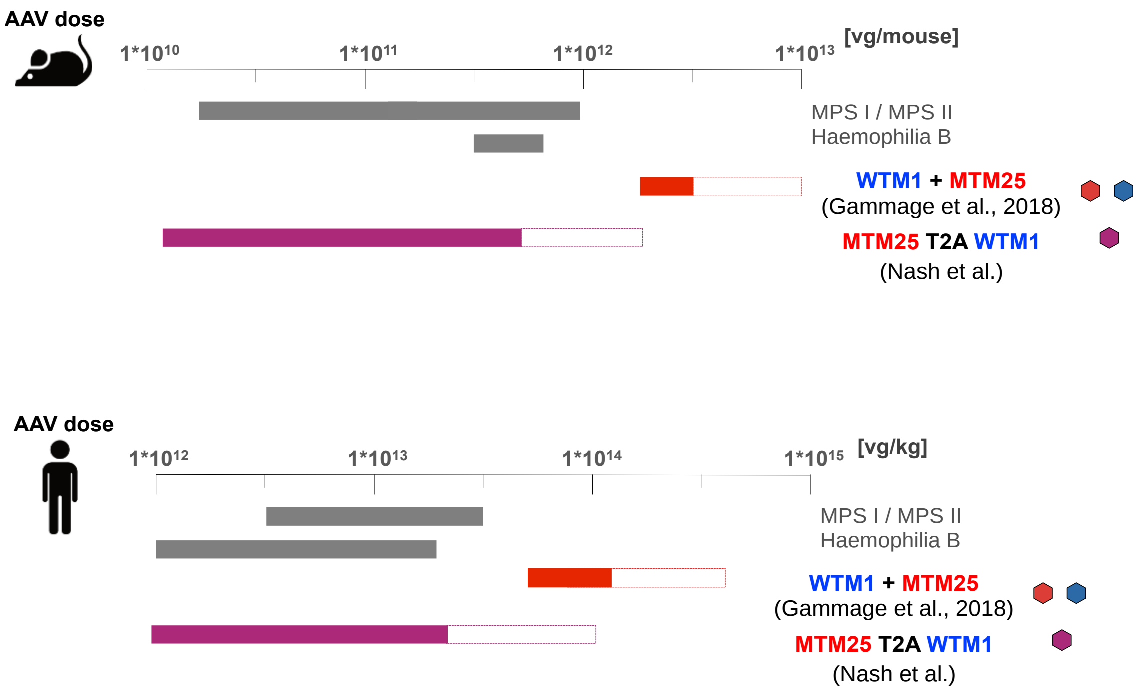

Appendix Figure S7. Comparison of AAV dosages in murine mtZFN experiments with human ZFN clinical trials

Schematic showing potential translatability of current tandem administration experiment to human clinical trials. Equivalent viral doses between mice and humans shown as range bars assuming 20-gram mice. Grey boxes denote human clinical trials using rAAVs, red box shows previous work performed by Gammage et al. and purple box shows dose ranges used in current work. Red dose ranges with no fill represent doses where heteroplasmy shifting was documented, but where significant depletions of mtCN occurred.

Appendix Supplementary Data

Alignment of MTM25(+)-T2A-WTM1(-) nucleotide sequence with MTM25-T2A-WTM1 after re-coding of WTM1. N.B. WTM1 FLAG tag replaced with HA during re-coding.

```
MTM25(+)-T2A-WTM1(-)
MTM25(+)-T2A-WTM1(-)_recoded

ATGTTGGGGTTTGTGGGTCGGGTGGCCGCTGCTCCGGCCTCCGGGGCCTTGCGGAGACTC      60
ATGTTGGGGTTTGTGGGTCGGGTGGCCGCTGCTCCGGCCTCCGGGGCCTTGCGGAGACTC      60
************************************************************

ACCCCTTCAGCGTCGCTGCCCCCAGCTCAGCTCTTACTGCGGGCCGCTCCGACGGCGGTC      120
ACCCCTTCAGCGTCGCTGCCCCCAGCTCAGCTCTTACTGCGGGCCGCTCCGACGGCGGTC      120
************************************************************

CATCCTGTCAGGGACTATGCGGCGCAATACCCCTACGACGTGCCCGACTACGCCGTGGAT      180
CATCCTGTCAGGGACTATGCGGCGCAATACCCCTACGACGTGCCCGACTACGCCGTGGAT      180
************************************************************

GAAATGACCAAAAAGTTCGGCACGCTCACCATTCACGACACCGAAAAGGCCGCCGAATTC      240
GAAATGACCAAAAAGTTCGGCACGCTCACCATTCACGACACCGAAAAGGCCGCCGAATTC      240
************************************************************

ATGGCTGAGAGGCCCTTCCAGTGTCGAATCTGCATGCGTAACTTCAGTGGCAACACCGGC      300
ATGGCTGAGAGGCCCTTCCAGTGTCGAATCTGCATGCGTAACTTCAGTGGCAACACCGGC      300
************************************************************

CTGAACTGTCACATCCGCACCCACACCGGCGAGAAGCCTTTTGCCTGTGACATTTGTGGG      360
CTGAACTGTCACATCCGCACCCACACCGGCGAGAAGCCTTTTGCCTGTGACATTTGTGGG      360
************************************************************

AGGAAATTTGCCGACCGCTCCAACCTGACCCGCCATACCAAGATACACACGCATCCCAGG      420
AGGAAATTTGCCGACCGCTCCAACCTGACCCGCCATACCAAGATACACACGCATCCCAGG      420
************************************************************

GCACCTATTCCCAAGCCCTTCCAGTGTCGAATCTGCATGCGTAACTTCAGTCAGTCCGGC      480
GCACCTATTCCCAAGCCCTTCCAGTGTCGAATCTGCATGCGTAACTTCAGTCAGTCCGGC      480
************************************************************

TCCCTGACCCGCCACATCCGCACCCACACCGGCGAGAAGCCTTTTGCCTGTGACATTTGT      540
TCCCTGACCCGCCACATCCGCACCCACACCGGCGAGAAGCCTTTTGCCTGTGACATTTGT      540
************************************************************

GGGAGGAAATTTGCCCACAAGTCCGCCCGCGCCGCCCATACCAAGATACACACGGGATCT      600
GGGAGGAAATTTGCCCACAAGTCCGCCCGCGCCGCCCATACCAAGATACACACGGGATCT      600

************************************************************

CAGAAGCCCTTCCAGTGTCGAATCTGCATGCGTAACTTCAGTCGCTCCGACCACCTGTCC      660
CAGAAGCCCTTCCAGTGTCGAATCTGCATGCGTAACTTCAGTCGCTCCGACCACCTGTCC      660
************************************************************

GCCCACATCCGCACCCACACCGGCGAGAAGCCTTTTGCCTGTGACATTTGTGGGAGGAAA      720
```

```
GCCCACATCCGCACCCACACCGGCGAGAAGCCTTTTGCCTGTGACATTTGTGGGAGGAAA      720
************************************************************

TTTGCCCAGCACGGCTCCCTGGCCTCCCATACCAAGATACACCTGCGGGGATCCCAGCTG      780
TTTGCCCAGCACGGCTCCCTGGCCTCCCATACCAAGATACACCTGCGGGGATCCCAGCTG      780
************************************************************

GTGAAGAGCGAGCTGGAGGAGAAGAAGTCCGAGCTGCGGCACAAGCTGAAGTACGTGCCC      840
GTGAAGAGCGAGCTGGAGGAGAAGAAGTCCGAGCTGCGGCACAAGCTGAAGTACGTGCCC      840
************************************************************

CACGAGTACATCGAGCTGATCGAGATCGCCAGGAACAGCACCCAGGACCGCATCCTGGAG      900
CACGAGTACATCGAGCTGATCGAGATCGCCAGGAACAGCACCCAGGACCGCATCCTGGAG      900
************************************************************

ATGAAGGTGATGGAGTTCTTCATGAAGGTGTACGGCTACAGGGGAAAGCACCTGGGCGGA      960
ATGAAGGTGATGGAGTTCTTCATGAAGGTGTACGGCTACAGGGGAAAGCACCTGGGCGGA      960
************************************************************

AGCAGAAAGCCTGACGGCGCCATCTATACAGTGGGCAGCCCCATCGATTACGGCGTGATC      1020
AGCAGAAAGCCTGACGGCGCCATCTATACAGTGGGCAGCCCCATCGATTACGGCGTGATC      1020
************************************************************

GTGGACACAAAGGCCTACAGCGGCGGCTACAATCTGCCTATCGGCCAGGCCGACGAGATG      1080
GTGGACACAAAGGCCTACAGCGGCGGCTACAATCTGCCTATCGGCCAGGCCGACGAGATG      1080
************************************************************

CAGAGATACGTGAAGGAGAACCAGACCCGGAATAAGCACATCAACCCCAACGAGTGGTGG      1140
CAGAGATACGTGAAGGAGAACCAGACCCGGAATAAGCACATCAACCCCAACGAGTGGTGG      1140
************************************************************

AAGGTGTACCCTAGCAGCGTGACCGAGTTCAAGTTCCTGTTCGTGAGCGGCCACTTCAAG      1200
AAGGTGTACCCTAGCAGCGTGACCGAGTTCAAGTTCCTGTTCGTGAGCGGCCACTTCAAG      1200
************************************************************

GGCAACTACAAGGCCCAGCTGACCAGGCTGAACCGCAAGACCAACTGCAATGGCGCCGTG      1260
GGCAACTACAAGGCCCAGCTGACCAGGCTGAACCGCAAGACCAACTGCAATGGCGCCGTG      1260
************************************************************

CTGAGCGTGGAGGAGCTGCTGATCGGCGGCGAGATGATCAAAGCCGGCACCCTGACACTG      1320
CTGAGCGTGGAGGAGCTGCTGATCGGCGGCGAGATGATCAAAGCCGGCACCCTGACACTG      1320
************************************************************

GAGGAGGTGCGGCGCAAGTTCAACAACGGCGAGATCAACTTCCTCGAGGAAGGAAGGGGC      1380
GAGGAGGTGCGGCGCAAGTTCAACAACGGCGAGATCAACTTCCTCGAGGAAGGGAGAGGA      1380
***************************************************** ** **

TCTTTGCTTACTTGTGGAGATGTTGAGGAAAATCCAGGACCCATGTTGGGGTTTGTGGGT      1440
TCTCTGCTTACTTGCGGCGATGTAGAGGAAAACCCCGGACCCATGCTCGGTTTCGTAGGC      1440
*** ********** ** ***** ******** ** ********* * ** ** ** **

CGGGTGGCCGCTGCTCCGGCCTCCGGGGCCTTGCGGAGACTCACCCCTTCAGCGTCGCTG      1500
CGCGTCGCTGCCGCACCAGCTTCAGGTGCACTCCGCCGATTGACACCCAGCGCAAGCCTT      1500
** ** ** ** ** ** ** ** ** **  * **  ** * ** **    **    **
```

```
CCCCCAGCTCAGCTCTTACTGCGGGCCGCTCCGACGGCGGTCCATCCTGTCAGGGACTAT      1560
CCTCCCGCACAGTTGTTGCTCCGAGCTGCCCCCACCGCCGTTCACCCCGTGCGAGATTAC      1560
** ** ** *** * ** ** ** ** ** ** ** ** ** ** ** **  * ** **

GCGGCGCAAGATTA---CAAGGACGACGATGACAAGGTGGATGAAATGACCAAAAAGTTC      1617
GCAGCTCAGTATCCTTATGATGTCCCTGATTATGCTGTAGACGAGATGACAAAGAAATTT      1620
** ** **  **       * * *   *** *    ** ** ** ***** ** ** **

GGCACGCTCACCATTCACGACACCGAAAAGGCCGCCGAATTCATGGCTGAGAGGCCCTTC      1677
GGAACTTTGACAATACATGATACAGAGAAAGCTGCAGAATTCATGGCCGAACGCCCTTTT      1680
** **  * ** ** ** ** ** ** ** ** ** *********** **  * ** **

CAGTGTCGAATCTGCATGCGTAACTTCAGTCTGCCGCACCACCTGGAGCAGCACATCCGC      1737
CAATGCCGGATTTGTATGAGAAATTTTTCTCTTCCTCACCACCTGGAGCAACATATTAGG      1740
** ** ** ** ** *** * ** **   *** ** ************* ** **  *

ACCCACACCGGCGAGAAGCCTTTTGCCTGTGACATTTGTGGGAGGAAATTTGCCCGCAAC      1797
ACACATACTGGGGAAAAACCCTTCGCTTGCGATATCTGCGGACGCAAGTTCGCTCGGAAT      1800
** ** ** ** ** ** ** ** ** ** ** ** ** **  * ** ** ** ** **

GCCTCCCGCACCCGCCATACCAAGATACACACGGGCAGCCAAAAGCCCTTCCAGTGTCGA      1857
GCTTCCCGCACTCGACACACAAAAATCCATACAGGGTCCCAGAAGCCATTCCAATGCAGG      1860
** ******** ** ** ** ** ** ** ** **   *** ***** ***** **  *

ATCTGCATGCGTAAGTTTGCCTACACCTACTCCCTGTCCGAGCATACCAAGATACACACG      1917
ATCTGCATGAGAAAGTTCGCATACACCTACTCTCTCTCTGAGCATACTAAAATTCACACT      1920
********* * ***** ** *********** ** ** ******** ** ** *****

GGCGAGAAGCCCTTCCAGTGTCGAATCTGCATGCGTAACTTCAGTCAGTCCGCCAACCGC      1977
GGGGAAAAACCATTTCAATGCAGAATATGTATGCGAAATTTCTCCCAGAGTGCTAATCGG      1980
** ** ** ** ** ** **  **** ** ***** ** ***   ***   ** ** **

ACCACCCACATCCGCACCCACACCGGCGAGAAGCCTTTTGCCTGTGACATTTGTGGGAGG      2037
ACCACTCATATTCGAACACATACAGGAGAAAAACCCTTCGCTTGCGATATCTGCGGACGA      2040
***** ** ** ** ** ** ** ** ** ** ** ** ** ** ** ** ** **  *

AAATTTGCCCACCGCTCCTCCCTGCGCCGCCATACCAAGATACACCTGCGGGGATCCCAG      2097
AAGTTCGCTCATAGGAGTAGCCTCCGCCGCCACACAAAAATCCATCTTCGCGGATCCCAA      2100
** ** ** **  *      *** ******** ** ** ** ** ** ** *******

CTGGTGAAGAGCGAGCTGGAGGAGAAGAAGTCCGAGCTGCGGCACAAGCTGAAGTACGTG      2157
CTTGTTAAATCAGAACTCGAAGAAAAAAAAAGCGAGCTACGCCATAAACTCAAATATGTA      2160
** ** **    ** ** ** ** ** **   ****** ** ** ** ** ** ** **

CCCCACGAGTACATCGAGCTGATCGAGATCGCCAGGAACAGCACCCAGGACCGCATCCTG      2217
CCTCATGAATATATTGAATTAATTGAAATTGCAAGAAATAGTACACAAGATCGAATTTTG      2220
** ** ** ** ** **  * ** ** ** ** ** ** ** ** ** ** ** **  **

GAGATGAAGGTGATGGAGTTCTTCATGAAGGTGTACGGCTACAGGGGAAAGCACCTGGGC      2277
GAAATGAAAGTCATGGAATTTTTTATGAAAGTATATGGTTACCGCGGCAAACATCTTGGA      2280
** ***** ** ***** ** ** ***** ** ** ** *** * ** ** ** ** **

GGAAGCAGAAAGCCTGACGGCGCCATCTATACAGTGGGCAGCCCCATCGATTACGGCGTG      2337
GGATCAAGGAAACCAGATGGGGCAATTTACACTGTTGGGAGTCCTATAGACTACGGGGTC      2340
***   ** ** ** ** ** ** ** ** ** ** ** ** ** ** ** ***** **
```

```
ATCGTGGACACAAAGGCCTACAGCGGCGGCTACAATCTGCCTATCGGCCAGGCCGACGAG      2397
ATTGTCGATACCAAAGCTTATTCTGGAGGGTATAACCTTCCCATTGGTCAAGCTGATGAA      2400
** ** ** ** ** ** **    ** ** ** ** ** ** ** ** ** ** ** **

ATGGAGAGATACGTGGAGGAGAACCAGACCCGGGATAAGCACCTGAACCCCAACGAGTGG      2457
ATGGAGCGCTATGTAGAAGAAAATCAAACAAGAGACAAACATCTTAACCCTAATGAATGG      2460
****** * ** ** ** ** ** ** **  * ** ** ** ** ***** ** ** ***

TGGAAGGTGTACCCTAGCAGCGTGACCGAGTTCAAGTTCCTGTTCGTGAGCGGCCACTTC      2517
TGGAAAGTCTATCCCAGTTCTGTTACTGAATTTAAATTTCTCTTTGTCTCTGGACATTTT      2520
***** ** ** ** **    ** ** ** ** ** ** ** ** **    ** ** **


AAGGGCAACTACAAGGCCCAGCTGACCAGGCTGAACCACATCACCAACTGCAATGGCGCC      2577
AAAGGAAATTATAAAGCTCAACTCACAAGATTAAATCATATAACAAATTGTAACGGTGCT      2580
** ** ** ** ** ** ** ** ** **  * ** ** ** ** ** ** ** ** **

GTGCTGAGCGTGGAGGAGCTGCTGATCGGCGGCGAGATGATCAAAGCCGGCACCCTGACA      2637
GTACTCTCAGTCGAAGAACTCCTCATTGGAGGTGAAATGATAAAGGCTGGAACACTCACC      2640
** **    ** ** ** ** ** ** ** ** ** ***** ** ** ** ** ** **

CTGGAGGAGGTGCGGCGCAAGTTCAACAACGGCGAGATCAACTTC   2682
CTCGAAGAAGTTCGCCGAAAATTTAATAATGGGGAAATTAATTTT   2685
** ** ** ** ** ** ** ** ** ** ** ** ** ** **
```
