# Supplementary figures and images for: Clinically translatable mitochondrial gene therapy in muscle using tandem mtZFN architecture
Source: EMBO Mol Med. 2025 Apr 9;17(6):1222–37. doi: 10.1038/s44321-025-00231-5 (PMC12163086; doi:10.1038/s44321-025-00231-5)

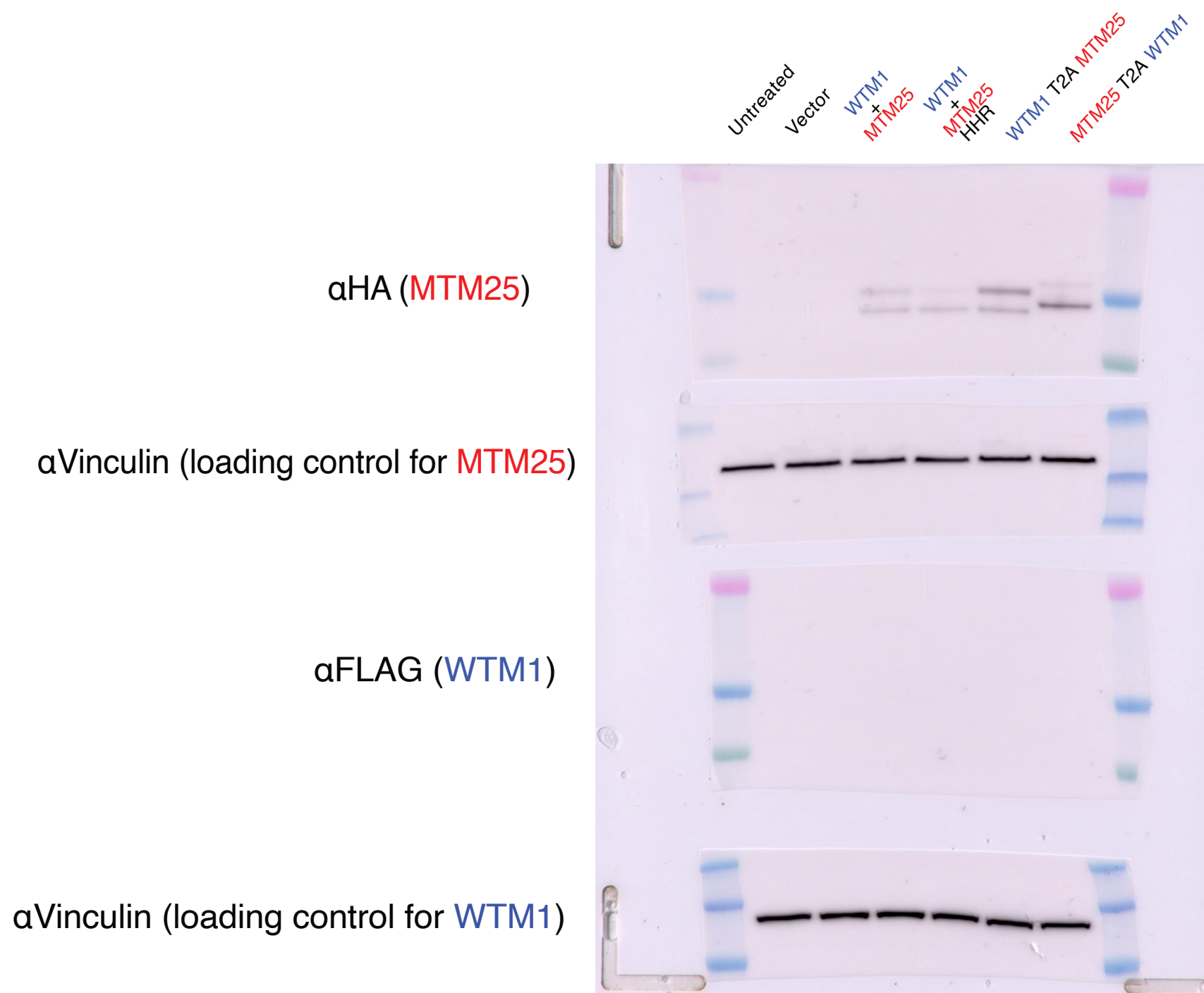

**Loading dye  
legend:**

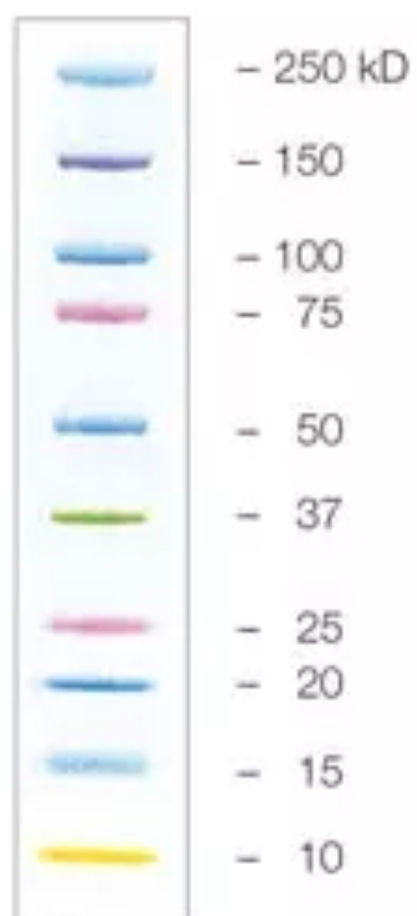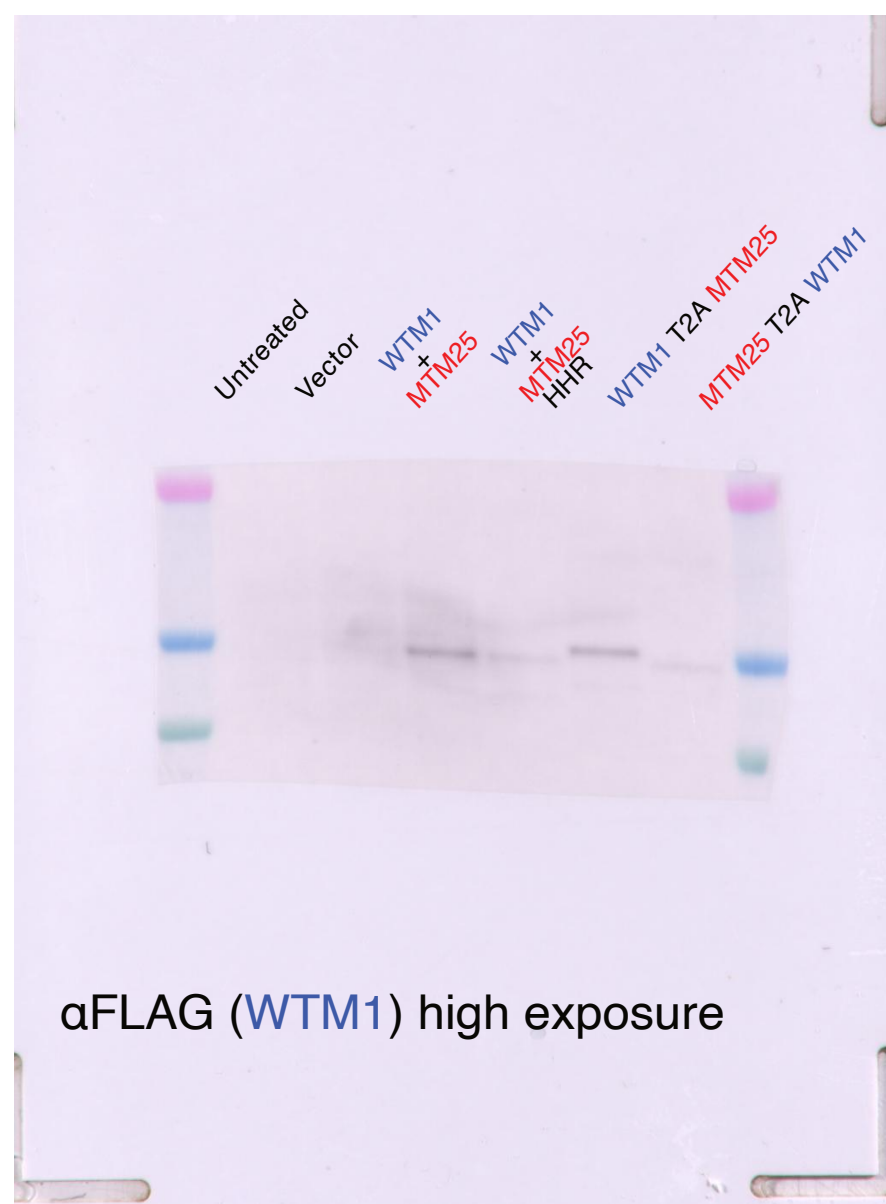

Supplement: Supplementary file 3 — Source data Fig. 2 [file 44321_2025_231_MOESM3_ESM.zip › Figure 2/2A/2A.pdf]
